# Invariance of the WHO violence against women instrument among Kenyan adolescent girls and young women: Bayesian psychometric modeling

**DOI:** 10.1371/journal.pone.0258651

**Published:** 2021-10-15

**Authors:** Benedict O. Orindi, Abdhalah Ziraba, Luk Bruyneel, Sian Floyd, Emmanuel Lesaffre

**Affiliations:** 1 Kenya Medical Research Institute-Wellcome Trust Research Programme, Kilifi, Kenya; 2 Department of Public Health and Primary Care, Leuven Biostatistics and Statistical Bioinformatics Centre, KU Leuven, Leuven, Belgium; 3 African Population and Health Research Center, Nairobi, Kenya; 4 Department of Public Health and Primary Care, Leuven Institute for Healthcare Policy, KU Leuven, Leuven, Belgium; 5 Faculty of Epidemiology and Population Health, London School of Hygiene and Tropical Medicine, London, United Kingdom; Population Council, INDIA

## Abstract

**Introduction:**

To make valid comparisons across groups, a measurement instrument needs to be measurement invariant across those groups. The present study evaluates measurement invariance for experience of violence among adolescent girls and young women (AGYW) in two informal settlements in Nairobi, Kenya.

**Methods:**

We used survey data collected from 1,081 AGYW aged 15–22 years from two Nairobi’s informal settlements of Korogocho (*n* = 617) and Viwandani (*n* = 464) in 2017 through DREAMS (an initiative aimed at reducing HIV incidence among AGYW with a core package of evidence-based interventions) impact evaluation project. Experience of violence was measured using the 15-item WHO’s violence against women instrument, and factorial (non)invariance assessed within exploratory structural equation modeling (ESEM) framework. Cross-group measurement invariance was assessed using Bayesian Multiple Indicator Multiple Causes (MIMIC) model across site, age groups, self-reported invitation to participate in DREAMS, marital status, currently in school, education level, religion, ethnic groups, ever had sex, slept hungry at night past 4 weeks, and wealth index.

**Results:**

The mean and median ages of the AGYW were 17.9 years and 17 years, respectively. About 59% reported having had sex and 58% of AGYW were in school. The percentage reporting each act of violence varied from 1.6% (“attacked you with a weapon”) to 26.5% (“insult you or make you feel bad about yourself”). About 44% (n = 474) of participants experienced ≥1 acts of violence, and 2.7% (n = 29) experienced at least half of the 15 acts. The structure underlying the 15 items was configurally similar to that proposed by WHO, with three factors reflecting either psychological, physical, or sexual violence. Noninvariance was detected for five items—spread across the three domains. Three of five items showed noninvariance only for sleeping hungry at night in the past 4 weeks. As the majority of items did not show evidence of noninvariance, differences in latent mean scores likely reflect actual differences and may not be attributable to measurement artifacts.

**Conclusions:**

Using state-of-the-art statistical techniques on a widely used instrument for measuring exposure to violence among women, this study provides support for the subscales of psychological, physical and sexual violence in a Kenyan AGYW population. The instrument supports comparisons across groups within this population. This is crucial when comparing violence against girls/women prevalence rates and to understand challenges and exchange strategies to reduce abuse or violence experienced by AGYW, or women in general.

## Introduction

Violence against women was put high on the agenda as an important public health problem and human rights abuse at key international conferences in the 1990s. The Fourth World Conference on Women held in Beijing in 1995 [1], in particular, established a strategic objective to study the causes and consequences of violence against women and the efficacy of preventive measures; and to encourage promotion of research on this subject. Prevalence studies on violence against women perpetrated by (intimate) male partners have since grown considerably, but with variability in figures reported across settings [2–10]. Whereas these differences may correspond to the actual differences in the magnitude of the problem in different settings, they may be due to study design and/or methodological differences (such as questionnaire content and questionnaire administration methods), or could be a reflection of cultural differences. Such discrepancies would limit meaningful comparisons among diverse settings.

To fill this gap, the “multi-country study on women’s health and domestic violence against women” was developed by the World Health Organization (WHO) [6]). The study tool included the violence against women (VAW) survey instrument to measure primarily violence by intimate partners experienced by women, especially physical, psychological and sexual violence. The WHO VAW instrument was crafted from the Conflict Tactics Scale (CTS) [11, 12], and followed a long process of discussion and consultation with technical experts in specific areas, largely because of the special ethical care the topic demands [6].

Several studies have since reported good internal consistency/reliability and validity of the instrument [4, 13–16].

Of critical importance is whether the instrument can be used among individuals with different characteristics or at different time points. If the instrument behaves differently across subgroups of the population, such as adolescent girls and young women, measurement biases could occur, resulting into invalid (or inappropriate) comparisons and interpretations that are not meaningful. In practice, these assumptions can be assessed through a statistical assessment of measurement invariance–also referred to as measurement equivalence [17, 18]. Different types of invariance can be distinguished. Configural invariance requires equality in terms of dimension (i.e., number of factors) and pattern (i.e., items within each factor) across groups. This type of invariance indicates similarity of concepts measured across groups. Metric invariance implies corresponding factors have the same meaning across groups; that is, equal factor loadings across groups. Scalar invariance implies equality in the meaning of the dimensions (i.e., equality of the factor loadings), and the levels of the underlying items (i.e., intercepts or thresholds) across groups. Scalar invariance is a necessary condition for meaningful comparison of group means [17–20]. Only a limited number of studies have rigorously evaluated this aspect of the WHO VAW instrument. Ribeiro et al. [21] assessed configural invariance and invariance of the factor loadings, but did not evaluate scalar invariance. In another study in two Brazilian municipalities, Ribeiro et al. [15] used confirmatory factor analysis (CFA) to investigate whether violence is a uni-or-multidimensional construct. They did not evaluate any cross-group measurement invariance. Other studies used either exploratory factor analysis (EFA) [16], or principal component analysis (PCA), which do not permit assessment of invariance [13, 14].

In this study we investigate whether (1) the factorial structure of the WHO VAW instrument for the adolescent girls and young women (AGYW) in Nairobi slums is configurally invariant [22] with the WHO’s classification [6], and (2) there is any evidence of cross-group invariance in experience of violence for important covariates; that is, whether the sub-populations of AGYW attribute the same meaning to the dimensions and the levels of the underlying items.

## Methods

### Study design, setting and sample

We analyzed primary data from the DREAMS IE (Determined, Resilient, Empowered, AIDS-free, Mentored, and Safe women Impact Evaluation) study. DREAMS is an initiative of PEPFAR (the US President’s Emergency Plan for AIDS Relief) to reduce new HIV infections among the most vulnerable AGYW in areas identified as ‘hot-spots’ with high HIV burden. DREAMS is using an HIV prevention package, being delivered “at scale”, to target the multiple sources of risks that AGYW face: behavioural, biological and structural (see [23]). DREAMS IE is a three-year project (2017–2019) with the objectives to: 1) measure whether HIV-related outcomes change due to DREAMS interventions at a population level; 2) explore the pathways of protection by which DREAMS interventions influence the lives of young women and ultimately their risk for HIV; and 3) assess the extent to which the DREAMS interventions are delivered as intended. In Nairobi (Kenya), the African Population and Health Research Center (APHRC) partnered with the London School of Hygiene and Tropical Medicine (LSHTM) to evaluate the impact of the DREAMS Initiative in two informal settlements of Korogocho and Viwandani. The full study protocol has been published elsewhere [24]. We focused on the AGYW survey component, in which a cohort of randomly selected girls/young women were enrolled during 2017 and followed prospectively, at ~12 and ~24 months. The present analysis uses baseline data collected from Nairobi on 1,081 AGYW (Korogocho, *n* = 617; Viwandani, *n* = 464) aged 15–22 years in 2017.

### Measures

Experience of violence was measured using the WHO’s VAW survey instrument [6]. The 15 items are listed in Box 1. The questions were like “Has any male ever done any of the following things to you in the past 12 months?”. The questions had two response options of “yes” (= 1) or “no” (= 0). The World Health Organization classifies the questions into three dimensions, i.e. psychological/emotional violence (items 1–3), physical violence (items 4–11), and sexual violence (items 12–15) [6]. Data were collected electronically using face-to-face interviews by forty-two carefully selected, properly trained and experienced field interviewers who were also well conversant with the study area. The tool was piloted and adjustments were made where needed.

Box 1. The 15 items of the World Health Organization Violence Against Women questionnaire used to measure experience of violence among AGYW in the DREAMS study.*

**Table:** 

**Items**
1. Say or do something to humiliate you in front of others?
2. Threaten to hurt or harm you or someone close to you?
3. Insult you or make you feel bad about yourself?
4. Push you, shake you, or throw something at you?
5. Slap you?
6. Twist your arm or pull your hair?
7. Punch you with his fist or something that could hurt you?
8. Kick you, drag you, or beat you up?
9. Try to choke you or burn you on purpose?
10. Threatened to attack you with a knife or other weapon?
11. Attacked you with a weapon?
12. Touched you in a sexual way (e.g. kissing, grabbing, or fondling), when you did not want them to?
13. Try to have sexual intercourse with you when you did not want to but did not succeed?
14. Physically forced you to have sexual intercourse even when you did not want to?
15. Forced you to perform sexual acts when you did not want to?

* The questions were like “Has any male ever done any of the following things to you in the past 12 months?”. The questions had two response options of “yes” (= 1) or “no” (= 0).

### Statistical methods

#### Factorial structure assessment

To assess whether the structure of the VAW instrument proposed by the WHO, with three dimensions, can be replicated in the Kenyan AGYW population (i.e. configurally invariant), we adopted the exploratory structural equation modeling (ESEM) framework of Asparouhov and Muthén [25]. ESEM is a more recent technique which, in addition to or instead of a CFA measurement model, allows an EFA measurement model with factor loading matrix rotations to be used in a structural equation model [25, 26]. The ESEM was likelihood-based using a limited-information weighted least squares estimation method with a Geomin rotation criterion equal to 0.001 (the 0.001 value is to improve the shape of the rotation function, so that it is easier to minimize and to reduce the number of local solutions, with larger values being used for models with more factors). To assess the fit of the ESEM model, we considered two indices which are functions of the likelihood ratio statistic, i.e., the comparative fit index (CFI) [27] and the Tucker-Lewis index (TLI) [28] and two indices based on how well a given model approximates the true model, i.e., the root mean square error of approximation (RMSEA) [29] and the weighted root-mean-square residual (WRMR). CFI- and TLI-values of at least 0.95, RMSEA values less than 0.06, and WRMR less than 1.0 are indicative of a good fit [30, 31].

#### Cross-group measurement invariance evaluation

To compare the latent means and to evaluate measurement invariance across groups, the MIMIC model [32, 33] was established by regressing the latent factors obtained from the ESEM model as well as the 15 items on covariates. If configurally invariant, the latent factors obtained from the ESEM model correspond with the 3 factors proposed by WHO. In our MIMIC model, a significant effect of a covariate on any of the violence latent variables (i.e., factors) indicates population heterogeneity (i.e., group differences on latent means). A significant direct effect of a covariate on any of the 15 items, over and above the indirect effect via the factors, indicates that the item is not invariant across the levels of that covariate (i.e., group differences on the indicator’s intercept or scalar noninvariance) [34]. That is, that particular item does not behave similarly across the levels of that covariate. Absence of such direct effect does not necessarily provide evidence of absence of noninvariance. Potential covariates for which invariance was assessed included self-reported invitation to participate in DREAMS (not-invited = 0, invited = 1), slum of residence (site: Korogocho = 0, Viwandani = 1), age at survey (15–17 years = 0, 18–22 years = 1), marital status (never married = 1, previously married/lived with partner = 2, currently married/living with partner = 3), currently in school (no = 0, yes = 1), educational level (none/incomplete primary = 1, complete primary = 2, incomplete secondary = 3, complete secondary = 4, tertiary = 5), religion (Muslim = 1, Christian = 2, and other = 3), ethnicity (Somali = 1, Kamba = 2, Kikuyu = 3, Kisii = 4, Luhya = 5, Luo = 6, Other = 7), ever had sex (no = 0, yes = 1), slept hungry at night in past 4 weeks (no = 0, yes = 1), and wealth index. Wealth index was constructed using principle component analysis (PCA) with input as indicator variables on ownership of household and individual assets/items (such as television, electricity, fridge, radio, bicycle, motorcycle, shoes, blanket, clothes, etc), household structure (i.e., floor, roof and wall material), and on household’s water supply and sanitation [35, 36]. It is common to split wealth index into quantiles. For our case, it was grouped into three categories of “poor” (= 1), “medium” (= 2), and “wealthy” (= 3).

We specified the MIMIC model using the latent variable parameterization via a probit link. The probit model assumes that for each dichotomous violence experience item, y, there is an underlying continuous, unobserved variable y* that follows a normal distribution with standard deviation unity. The exact continuous measurements of y* (which expresses the true experience of violence) are not available, but are related to the observed dichotomous variable *y* such that for y* > 0, violence experience is expressed on a manifest scale indicated by y = 1, and zero otherwise. Thus a linear regression for y* is equivalent to a probit regression for y (see e.g. Gelman and Hill [37] for more on latent variable parameterization). Details of the MIMIC model are provided in the S1 Appendix.

#### Selection of covariates for the assessment of direct effect on the items

We assume a probit model again. First, invitation to DREAMS, site and age adjusted model was run with one covariate at a time for each of the 15 items. Next, using a likelihood ratio test (LRT) all covariates significant at p≤0.10 in the first step above were included in a multivariable model. Finally, covariates found to be significant in the multivariable model at p≤0.05 (adjusted for invitation to DREAMS, site and age) were included in the direct effect of covariates part of the MIMIC model (i.e., the A part of the MIMIC model in equation 1 described in the S1 Appendix) for assessment of cross-group measurement (non)invariance. Invitation to DREAMS, site, and age were retained even if they were not significant as they were of interest to the research, but we also wished to correct for their impact. S2 Table summarizes the results of this exercise. All eleven covariates were included for assessment of group differences on latent means (i.e., in the B part of MIMIC model in equation 1 in the S1 Appendix).

Data management was performed using Stata v14.2 (StataCorp, College Station, TX) and all analyses were performed using M*plus* v7.4 [38]. We used a combination of frequentist and Bayesian approaches. For the variable selection described in the above paragraph, frequentist methods were used as they are considerably faster than the Bayesian methods. The MIMIC model was fit in a Bayesian framework (see e.g. Lesaffre and Lawson [39] for a full, pedagogical introduction to Bayesian inference) using the Bayesian structural equation modeling (BSEM) approach proposed by Muthén and Asparouhov [40]. We present standardized estimates. Full computational details, including model fit assessment is provided in the S1 Appendix.

### Ethical considerations

The study protocol, including informed consent and study tools, were reviewed and approved by the Observational Research Ethics Committee of the London School of Hygiene and Tropical Medicine (Ref 211 11835). Additionally, ethical approval was received from AMREF (ESRC P298/2016). Study participants also provided informed, written consent to participate in the study. For legal minors (i.e., those aged < 18 years), assent was obtained from the minor after the parent or guardian gave consent.

## Results

### Descriptive findings

**Fig 1:**
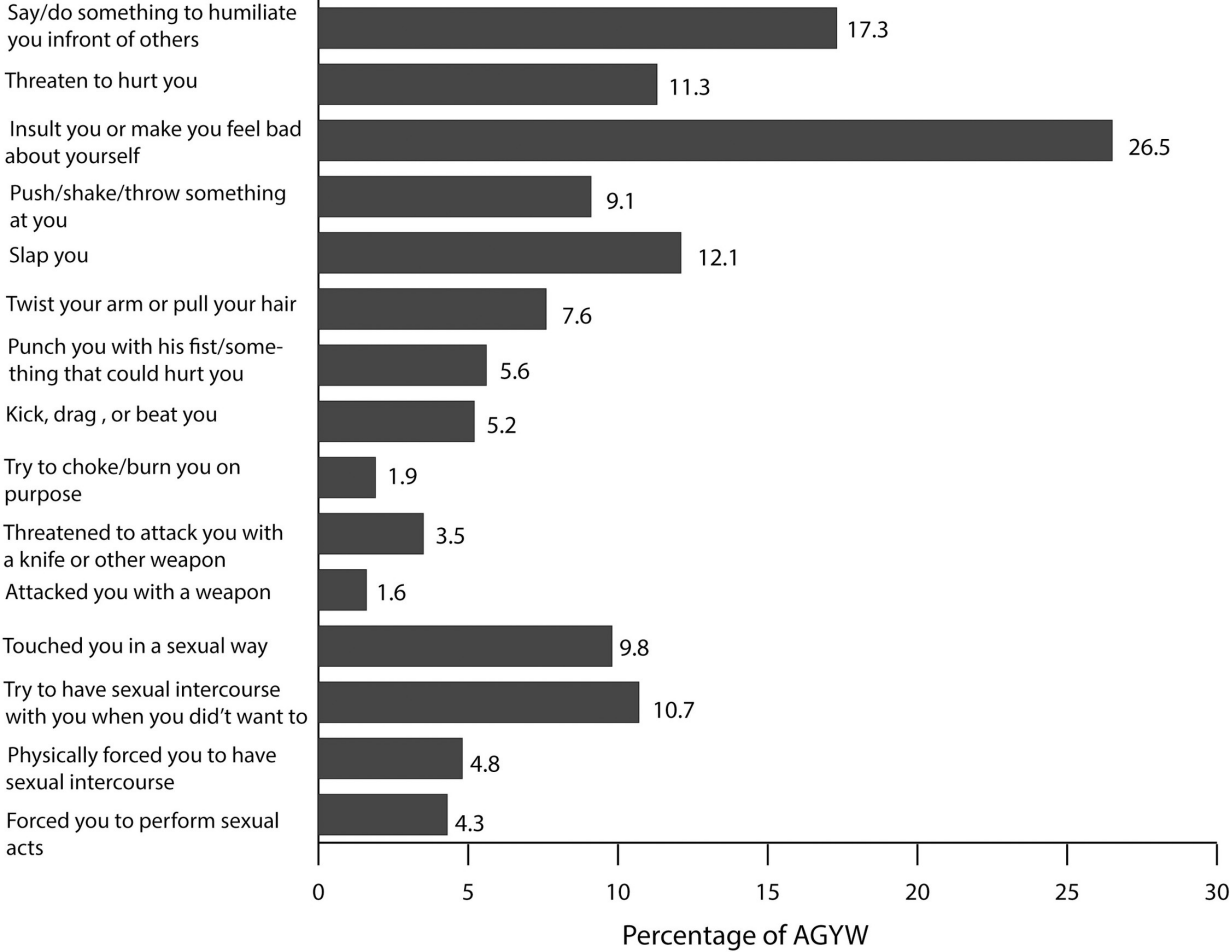
DREAMS survey data: Percent of AGYW reporting each of the 15 acts of violence.

The mean and median ages of the AGYW were 17.9 years and 17 years respectively. The majority had never been married (843/1081), were in school (625/1081), were Christians (917/1081), and had ever had sex (642/1081). The AGYW were from different ethnic groups. S1 Table shows the distribution of AGYW by socio-demographic characteristics. Fig 1 shows, for each of the 15 items, the percentage of AGYW who reported to have experienced violence in the past 12 months. It shows that the proportions ranged from a high of 26.5% for “insult you or make you feel bad about yourself”, to a low of 1.6% for “attacked you with a weapon”.

**Fig 2:**
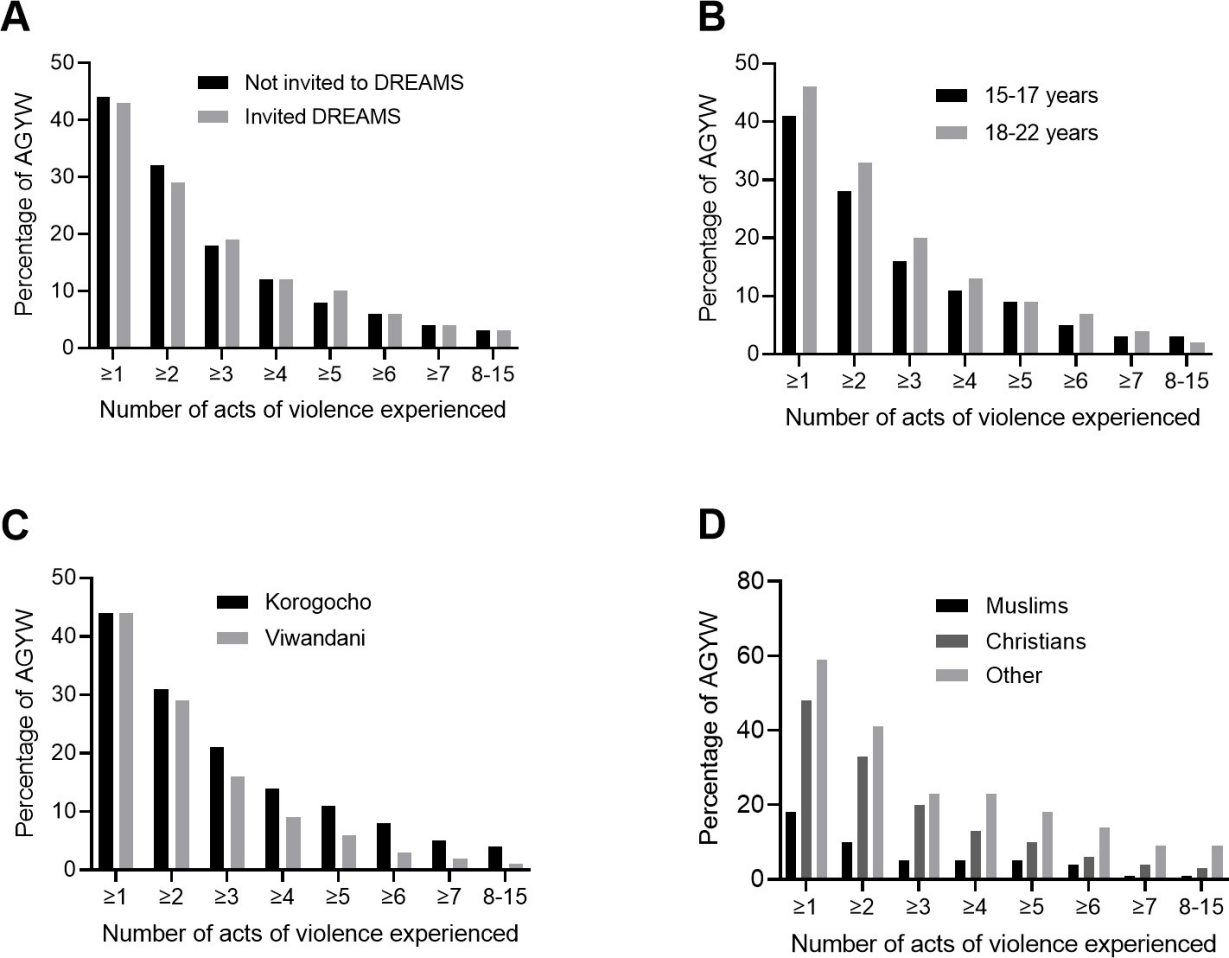
DREAMS survey data: Percentage of AGYW who experienced one or more violence acts, two or more acts, three or more acts, and so on up to 8 or more acts of violence, stratified by invitation to participate in DREAMS (A), age (B), site (C) and religion (D).

About 44% (n = 474) of the AGYW experienced at least one act of violence, 2.7% (n = 29) experienced at least half of the acts of violence, and 0.3% (n = 3) experienced 14 acts. No respondent experienced all 15 acts. Fig 2 shows the percentage of AGYW who experienced one act or more, two or more acts, three or more acts, and so on up to 8 or more acts, stratified by invitation to participate in DREAMS, age, site and religion. The numbers were similar between invited and non-invited, and between 15–17 year and 18–22 year olds experiencing at least 4 acts. The numbers were consistently higher for Korogocho than Viwandani; about 5% (n = 23) and 1% (n = 6) reported at least 8 items in Korogocho and Viwandani, respectively. These proportions also varied by religion.

### Factorial structure of the WHO VAW instrument for the Kenyan AGYW population

Goodness-of-fit indices for this study indicated the ESEM solution summarized the 15 items well (CFI = 0.996, TLI = 0.993, RMSEA = 0.026 (90% Cl: 0.017–0.034), WRMR = 0.634).

Table 1 shows the standardized factor loadings and factor correlations obtained from the ESEM model. The results verified the hypothesized factor structure. It can be seen that all the hypothesized major loadings were high (absolute value = 0.543–0.943). The factor loadings gave a clear and parsimonious interpretation of the factors in terms of psychological (the first 3 items), physical (items 4 through 11), and sexual violence (items 12 through 15). There were some cross-loadings (absolute value = 0.001–0.352)—suggesting the cross-loadings need not be restricted to zero (as is usually done in CFA using the frequentist approach to identify the model). As explained earlier, we took care of this in the Bayesian MIMIC model by allowing for cross-loadings using informative, small-variance priors. The factor correlations were medium to high and were positive, pointing to the fact that these three factors are measuring an underlying domain of experience of violence. The highest factor correlation was between psychological and physical violence factors. The smallest correlation was between psychological and sexual violence factors.

**Table 1:** DREAMS survey data: Exploratory structural equation modeling factor solution.

Item	Factor		
	Psychological	Physical	Sexual
1. Say or do something to humiliate you in front of others?	**0.886** *	0.001	-0.048
2. Threaten to hurt or harm you or someone close to you?	**0.543** *	0.288*	0.092
3. Insult you or make you feel bad about yourself?	**0.755** *	0.126	0.041
4 Push you, shake you, or throw something at you?	0.352*	**0.516** *	-0.021
5. Slap you?	0.086	**0.756** *	-0.038
6. Twist your arm or pull your hair?	0.044	**0.664** *	0.133
7. Punch you with his fist or something that could hurt you?	-0.007	**0.943** *	-0.061
8. Kick you, drag you, or beat you up?	0.040	**0.934** *	-0.047
9. Try to choke you or burn you on purpose?	-0.047	**0.873** *	0.004
10. Threatened to attack you with a knife or other weapon?	0.183	**0.663** *	0.019
11. Attacked you with a weapon?	-0.247	**0.816** *	0.143
12. Touched you in a sexual way (e.g. kissing, grabbing, or fondling), when you did not want them to?	0.099	0.228*	**0.644** *
13. Try to have sexual intercourse with you when you did not want to but did not succeed?	-0.037	0.278*	**0.752** *
14. Physically forced you to have sexual intercourse even when you did not want to?	0.020	0.052	**0.917** *
15. Forced you to perform sexual acts when you did not want to?	0.167	-0.017	**0.896** *
Factor correlations			
Psychological	1.000		
Physical	0.575*	1.000	
Sexual	0.446*	0.467*	1.000

Bold values indicate hypothesized major loadings;

* indicates values are significant at 5% level; ESEM, exploratory structural equation modeling.

In summary, the obtained factor structure was configurally similar to the one proposed by the WHO [6].

### Measurement invariance of the WHO VAW instrument across groups of AGYW

First, we observe that the Bayesian MIMIC model provided a good fit to the data (PPP-value = 0.468). Including the effects of the 11 covariates (i.e., specified in the *x*_*i*_ vector in equation 1 for B-coefficients in the MIMIC model described in the S1 Appendix) on the three violence factors as well as the effects of the covariates selected on the basis of a series of univariate models—as described in the Statistical methods section—on the 15 items (i.e., specified in the *x*_*i*_ vector in equation 1 for A-coefficients in the MIMIC model described in the S1 Appendix) did not undermine the hypothesized factor solution obtained from the ESEM model presented in Table 1 (see S3 Table). Factor correlations were, however, relatively higher than those from the ESEM model. We will come back to factor correlations in the Discussion section. Table 2 shows the effect of covariates on the latent mean scores for the three factors (i.e., B-coefficients in equation 1 in the S1 Appendix) as well as on the 15 items (A-coefficients in equation 1 in the S1 Appendix) from the MIMIC model. The left panel on the effects of covariates on factors shows the means for psychological violence factor were significantly higher among those who had ever had sex. For physical violence factor, the means were significantly lower among AGYW with complete secondary education, compared to those who never attended school or had incomplete primary level of education. For sexual violence factor, the means were significantly lower among those with complete primary education than those with no education or incomplete primary education, and among those in the medium wealth quantile. On average, sexual violence was less in ethnic groups other than Somali. The means of sexual violence factor were significantly higher among non-Muslims and girls/women who had ever had sex (compared to those who have never have sex).

**Table 2:** Bayesian MIMIC model: Effect of covariates on factors (B-coefficients) and on 15 experience of violence items (A-coefficients).

	Effect of covariates on factors (B-coefficients)			Effect of covariates on the 15 violence items (A-coefficients)														
Covariates	Psychological	Physical	Sexual	1	2	3	4	5	6	7	8	9	10	11	12	13	14	15
Invited to DREAMS (Ref: not invited)	-.005	-.021	-.088	-.039	.024	.012	.095	.040	.056	-.019	.012	-.055	-.047	-.134	.036	.041	-.066	.015
Site/slum (Ref: Korogocho)	-.006	-.207	.011	.020	-.008	.035	-.046	.024	.023	.050	-.002	.044	-.244*	-.074	.026	-.013	-.039	.030
Age (Ref: 15–17yrs)	.011	-.097	-.133	.033	.066	-.031	-.038	.057	-.108	.032	.039	-.159	-.028	.093	-.023	-.036	-.001	-.007
Marital/co-habitation status (Ref: never married)																		
Previously married/lived with partner	.090	.071	-.067	-.038	-.032	.016		.030										
Currently married/living with partner	-.184	-.020	-.096	.047	.006	.050		.116*										
Currently in school (no/yes. Ref: no)	-.059	-.090	-.017									-.139						
Educational level (Ref: None/incomplete primary)																		
Complete primary	-.103	-.103	-.133*					-.068										
Incomplete secondary	.022	-.134	-.076					-.135*										
Complete secondary	-.044	-.167*	-.036					-.062										
Tertiary: university/college/vocational	-.045	-.109	.032					-.036										
Religion (Ref: Muslim)																		
Christian	.208	.196	.578*						.058									
Other	.096	.101	.250*						-.057									
Ethnicity (Ref: Somali)																		
Kamba	.079	-.137	-.535*															
Kikuyu	.050	-.153	-.590*															
Kisii	.052	-.029	-.305*															
Luhya	.021	-.159	-.481*															
Luo	.062	-.081	-.458*															
Other	.002	-.049	-.266*															
Ever had sex (no/yes. Ref: no)	.133*	.090*	.372*					.071							-.020	-.034	.098	.001
Slept hungry at night past 4 weeks (no/yes. Ref: no)	.066	.052	.052	.090*	.100*		.032		.029				.068			.072	.073	.139*
Wealth quantile (Ref: Poor)																		
Medium	-.010	-.077	-.103*										-.215*					
Wealthy	.011	.002	-.060)										.033					

*Indicates 95% credibility interval does not contain zero. Full definitions of items 1–15 are listed in Box 1. Full estimation results are in the S4 and S5 Tables.

The right panel on the effect of covariates on the 15 violence items shows evidence of measurement noninvariance for five items: 1) Three items “Say or do something to humiliate you in front of others”, “Threaten to hurt or harm you or someone close to you”, and “Forced you to perform sexual acts when you did not want to” each had one significant direct effect, in the sense of the 95% Bayesian credibility interval not containing zero, from the covariate slept hungry at night past 4 weeks; 2) “Slap you” had two significant direct effects from the covariates marital/co-habitation status and education level; and 3) “Threatened to attack you with a knife or other weapon” had two significant direct effects from the covariates site and wealth quantile. The significant direct effects can be interpreted as follows, in the case of site as an example. For a given factor value, AGYW in Viwandani slum had a lower propensity of giving a yes answer to the question “Has any male ever threatened to attack you with a knife or other weapon in the past 12 months?” than those in Korogocho slum. We note that whereas other direct effects were not significant, small nonzero estimates were obtained.

In general, for most items we found no evidence of deviation from measurement invariance across groups of Kenyan AGYW. Thus, the instrument is invariant and practical terms and the differences in factor means could therefore be interpreted as actual differences.

## Discussion

The purpose of the present paper was to investigate the factorial structure and measurement invariance of the WHO VAW instrument among vulnerable Kenyan AGYW population. We rigorously evaluated measurement invariance of the WHO VAW instrument among AGYW Kenyan population in an urban slum setting. Using state-of-the-art statistical techniques, we obtained a 3-factor solution comprising psychological, physical, and sexual domains, configurally similar to the one proposed by World Health Organization [6]. Our findings of no indications of noninvariance for the WHO VAW instrument items with respect to marital status, education level, religion, ethnicity, and whether one had ever had sex implies the significant differences in their latent factor means need be interpreted as actual differences in magnitude of exposure to violence. That is, the instrument is equally reflective of the constructs of interest in all instances considered and the differences in the latent mean scores correspond to actual differences and may not be attributed to artifacts resulting from study design, methodological differences, or cultural differences. There was, however, evidence of noninvariance for items 1, 2, and 15 (with respect to slept hungry at night past 4 weeks), item 5 (for single levels of marital/cohabitation status and education level), and item 10 (for slum of residence and wealth quantile). Sass [41] provides guidelines on how to handle noninvariant items. That is, either 1) use invariant items only; or 2) apply a partial measurement invariance model; 3) assume that for the items with measurement noninvariance, the differences are too small to influence the results and proceed using all the items; or 4) simply avoid using the scale. He argues the third option is feasible when the degree of measurement noninvariance is minimal and the majority of items are invariant. For our case, we assumed this option as noninvariance was detected for five items only—spread across the three domains—for single levels of the covariates, with three of them being attributed to a single covariate of slept hungry at night past 4 weeks. We, however, note that in another study in Brazil using the same tool—although it did not evaluate measurement invariance—the authors reported a Heywood situation (i.e., a negative value for the residual variance) in a CFA model with respect to item 10 [15]. They resolved it by excluding the item from the CFA analysis. Thus, even as we assume the third option for the present study, the contents of item 10 need to be given careful attention.

Whereas our findings may permit comparison of Kenyan results across groups within the country, caution needs be exercised as there are other factors such as rural/urban residence, entire age continuum, etc, which we did not look at but may impact on how the tool performs. Consider residence as an example, the slum population is generally unique with different social challenges compared to their rural and urban non-slum counterparts.

Our finding of configural invariance of the factor structure in the Kenyan AGYW population alone does not, however, permit valid international comparisons [42, 43]. When interest is to make comparisons across countries, then it is important to ascertain that these measurements are invariant across countries. Such international studies often result into data that have a multilevel structure (e.g., data of girls/women clustered in countries). Multilevel factor analytic models can be applied to evaluate measurement invariance across the hierarchical levels of the study and across groups at those specific levels. Several authors have underscored the importance of assessment of cross-level invariance, including the fact that the meanings of the factors may differ across those hierarchical levels, that it easily allows for deeper understanding of differences across countries by allowing for inclusion of country-level variables (such as general income level or literacy level) in the model to explain potential country bias in survey items, and that one can evaluate how much of the common factor variance exist between countries and how much exist within countries [20, 44–48]. Heise and Kotsadam [49] recently used a multilevel model to investigate how macro-level factors impact women’s risk of intimate partner violence among 44 countries, but without evaluating cross-country invariance. Studies with univariate data can assess invariance across settings by including score-by-country interaction terms in the model as in standard differential functioning approaches.

The factor correlations from the likelihood-based ESEM were relatively smaller than the Bayesian factor correlations. The cross-loadings in the ESEM may contribute to the lower factor correlations as less correlations among the items need to go through the factors. However, the Bayesian factor correlations are not excessively high since the factors are expected to correlate to a substantial degree according to theory. These high correlations may also suggest a need to model a second-order factor(s) [50]. We advocate for further research to explore this.

We evaluated invariance across groups using MIMIC modeling within the BSEM framework. The MIMIC modeling approach is more parsimonious, allow smaller sample sizes, can accommodate several covariates simultaneously, and can allow for continuous covariates (e.g., age) as well as interactions. However, it only studies higher level invariance (i.e., intercept/threshold/scalar invariance). As we have demonstrated, a Bayesian framework offers a flexible approach that allows for comprehensive evaluation of measurement invariance, overcoming some challenges often experienced in a frequentist approach such as convergence/nonidentification. A discussion on this, and other related issues, can be found in Garret and Zeger [51], Muthén and Asparouhov [52] and Levy and Mislevy [53]. Other methods for studying measurement invariance have been developed. First, is multiple-group confirmatory factor analysis (MGCFA) [54, 55], involving running a set of increasingly constrained structural equation models (SEM) to test the nested forms of invariance. It is used when measurement invariance is tested with respect to a grouping variable (e.g., invited to DREAMS vs not invited, girls vs. boys). The second is alignment optimization [56], which replaces setting equality constraints with a procedure that is similar to rotation in exploratory factor analysis which looks for the ‘best’ solution. Third, is restricted factor analysis (RFA) [57, 58] which is similar to MIMIC analysis except that in MIMIC models, the covariates have causal effects on the latent factors, whereas in the RFA approach the covariates and latent factors are merely associated.

Two important methodological recommendations emerge from the present study. First, concerns model evaluation tools. In the present analyses we evaluated models based on posterior predictive checks (PPCs), as the only available tool in M*plus* for single-level SEM with categorical variables. Despite its importance to structural equation modeling (SEM), model evaluation remains underdeveloped for the BSEM. The PPP-value is a Bayesian tool for assessing goodness of fit available in popular software [59, 60]. Deviance information criteria (DIC) [61] is a generalization of frequentist Akaike information criteria (AIC) to choose between models, in which the model complexity penalty is determined using the deviance of the hypothesized model [60]. Recently, MK Cain and Z Zhang [62] evaluated PPP-value and DIC in a series of Monte Carlo simulation studies, but for continuous variables only. In another study, Hoofs and colleagues evaluated the Bayesian variant of the root mean square error of approximation (RMSEA) [63]. Zhang et al. [64] also introduced five variants of DIC as a model selection index for multilevel IRT models with dichotomous outcomes in WinBUGS. Second, concerns Bayesian variable selection (BVS) in SEMs. We selected covariates for which to study direct effects in the MIMIC model by first applying classical forward selection and backward elimination techniques on each of the 15 violence items. A number of approaches for BVS have been developed (see e.g. Lesaffre and Lawson [39], and Miller [65]). Extending these approaches to Bayesian SEMs will be the focus of our next paper.

In summary, meaningful comparisons across groups can be performed with confidence if measurement invariance is evaluated. While the present analysis has not detected noninvariance for a large number of covariates in this population, we hope researchers of violence against women/girls will find value in assessing measurement invariance as a way of addressing methodological issues in the study of violence against women/girls. This is especially important because the WHO violence against women instrument was built on the tradition of CTS, in the sense that respondents are asked questions about their experiences of specific acts of psychological, physical or sexual violence by a male (partner). Whereas asking such behaviourally specific questions encourages greater disclosure than requiring respondents to identify themselves as abused [66], the interpretations are dependent on subjective perceptions.

## Conclusions

Our findings support comparisons across groups, which is important when comparing violence against girls/women prevalence rates between groups to reduce abuse or violence experienced by girls/women perpetrated by male partners. Further studies examining invariance and other psychometric properties of the instrument—especially among AGYW—need to be conducted in other countries before comparing prevalence at the international level. This paper follows from a call by Sharpe [67] for an increase in papers that bridge knowledge from the statistical and psychometric community to researchers who apply these methods to their empirical data. We believe researchers concerned with instrumentation in other fields will also find merit in our work and apply it in their respective areas.

## Supporting information

S1 Appendix The multiple indicators multiple causes model specification and computational detail. (DOCX)

S1 Table DREAMS survey data. Distribution of AGYW by Socio-demographics.
(DOCX)

S2 Table DREAMS survey data. Selection of covariates for the direct effect assessment in the A coefficient. Data are LRT p-values.
(DOCX)

S3 Table DREAMS survey data. Bayesian MIMIC model: Confirmatory Factor Analysis.
(DOCX)

S4 Table Bayesian MIMIC model: Effect of covariates on factors (B-coefficients). (DOCX)

S5 Table Bayesian MIMIC model: Effect of covariates on 15 experience of violence Items (A-coefficients). (DOCX)

S1 Dataset De-identified data. (RAR)
