# Supplementary material for: Invariance of the WHO violence against women instrument among Kenyan adolescent girls and young women: Bayesian psychometric modeling
Source: PLoS One. 2021 Oct 15;16(10):e0258651. doi: 10.1371/journal.pone.0258651 (PMC8519454; doi:10.1371/journal.pone.0258651)
Supplement: S1 Appendix — (DOCX) [file pone.0258651.s001.docx]

**Supplementary Online Material**

Invariance of the WHO violence against women instrument among Kenyan adolescent girls and young women: Bayesian psychometric modeling

Benedict O Orindi^1,2,3*^, Abdhalah Ziraba^3^, Luk Bruyneel^4^, Sian Floyd^5^, Emmanuel Lesaffre^2^

**The Multiple Indicators Multiple Causes Model Specification**

We specify the MIMIC model as follows using the latent variable parameterization via a probit link (see e.g. Gelman and Hill [1] for more on latent variable parameterization):

$y_{i}^{*}=Ax_{i}+\Lambda\xi_{i}+\varepsilon_{i},$

$\xi_{i}=Bx_{i}+\delta_{i},$ (1)

$$\delta_{i}\sim N\left( 0, \Sigma\right), \varepsilon_{i} \sim N\left( 0, I_{p} \right), i=1,2,3,\ldots, n$$

where $y_{i}^{*}$ is a p × 1 vector of items for girl/woman $i$. It is the underlying continuous measure of the observed dichotomous experience of violence item $y_{i}$ such that for$y_{i}^{*}>0,$ violence experience is expressed on a manifest scale indicated by$y_{i}=1$, otherwise zero. The probit model assumes that the underlying latent variable$y_{i}^{*}$ follows a normal distribution with standard deviation one. Thus a linear regression for $y_{i}^{*}$ is equivalent to a probit regression for$y_{i}$. The $p\times r$ matrix A of the direct effects is associated with the $r\times1$ vector of covariates $x_{i}$ specified above, Further, $\Lambda$ is a $p\times q$ matrix of factor loadings (i.e., regression coefficients relating the common factor to the factor items, $\lambda_{s}$), $\xi_{i}$ is a $q\times1$ vector of latent factors distributed as $N(0,\Phi)$, $\varepsilon_{i}$ is a $p\times1$ random vector of error measurements and is independent of $\xi_{i}$, B is a $q\times r$ unknown parameter matrix associated with the vector of covariates $x_{i}$,$\delta_{i}$ is a $q\times1$ vector of residual errors. Cross-group (non)invariance is then assessed by studying the significance of the A-coefficients, i.e. a significant A-coefficient indicates that the item is non-invariant across the levels of that covariate. The effect of the covariates on the violence factors is assessed by studying the significance of B-coefficients in Model 1 above.

**Computational Details**

Data management was performed using Stata v14.2 (StataCorp, College Station, TX) and all analyses were performed using M*plus* v7.4 [2]. We used a combination of frequentist and Bayesian approaches. For the variable selection described in the above paragraph, frequentist methods were used as they are considerably faster than the Bayesian methods. The MIMIC model was fit in a Bayesian framework (see e.g. Lesaffre and Lawson [3] for a full, pedagogical introduction to Bayesian inference) using the Bayesian structural equation modeling (BSEM) approach proposed by Muthén and Asparouhov [4]. The choice of a Bayesian analysis needs some motivation. First, in a conventional frequentist approach freely estimating the cross-loadings and all direct effects of all the covariates on the 15 items would render the model non-identified because the $q^{2}$ restrictions (where $q$ is the number of factors) necessary to eliminate indeterminacies are absent [5]. The indeterminacies are resolved by fixing cross-loadings to zero, but this is—in many cases—overly stringent. Substantive theory does not dictate that the cross-loadings are exactly 0, just that they are low in magnitude. Second, we report here that we experienced computational challenges with either the direct effects of covariates on the items or the effect of covariates on the violence factors estimated in the MIMIC model using frequentist ESEM approach due to the low frequencies for items 9 and 11. The Bayesian approach overcomes these challenges by allowing easy imposition of “hard” constraints (e.g. a cross-loading equals 0) and “soft” constraints (e.g. a cross-loading is likely near 0) while preserving the core of the substantive theory by varying the prior variances. Replacing cross-loadings specifications of exact zeros with approximate zeros based on small-variance priors brings information into the analysis that avoids the non-identification problem [4]. It is not immediately clear how small the prior variances need to be to solve the indeterminacy. Increasing them too much can also lead to identification problem. So we vary them empirically. In our analysis, all item cross-loadings were given normal priors with zero mean and variance 0.02 (allow for small cross-loadings which provide a more flexible solution than fixing these cross-loadings to zero), while the direct effects of covariates on the 15 violence experience items were given normal priors with zero mean and variance 1. The *N* (0,1) prior variance was selected here because of three reasons: 1) Smaller prior variances produced worse model fit; 2) whereas same results were obtained when the variance was gradually increased up to 20, time to get convergence increased considerably; and 3) very large variances rendered the model non-identified. This is also in line with Gelman et al [6] in which weakly informative priors, such as *N* (0, 1), *N* (0, 5), and *N* (0, 20), are recommended for probit and logistic regression although preference is given there to priors based on the T-distribution and the Cauchy distribution. In addition, Asparouhov and Muthén [7] warned against the use of generic non-informative priors in factor analytic models with binary items. They argued that specifying priors that have unlimited uniform range for parameters that are on probit scale is not ideal as such priors induce skewed priors on probability scale. They encourage researchers to choose priors with a reasonable and finite range. Other than the cross-loadings and the direct effects, the default non-informative prior settings in M*plus* were not overridden [4]. The coefficients for the MIMIC model were standardized by setting the variances of the continuous latent variables to unity.

Two chains each having 200,000 Markov Chain Monte Carlo (MCMC) iterations were initiated. Thinning of one in 20 iterations was implemented. Convergence of all parameters was assessed using trace plots and the Brooks-Gelman-Rubin [8]. The mixing of MCMC chains (see S1 Fig) together with values of potential scale reduction factor (PSRF)*<*1.1 were considered indicative of no convergence problems.


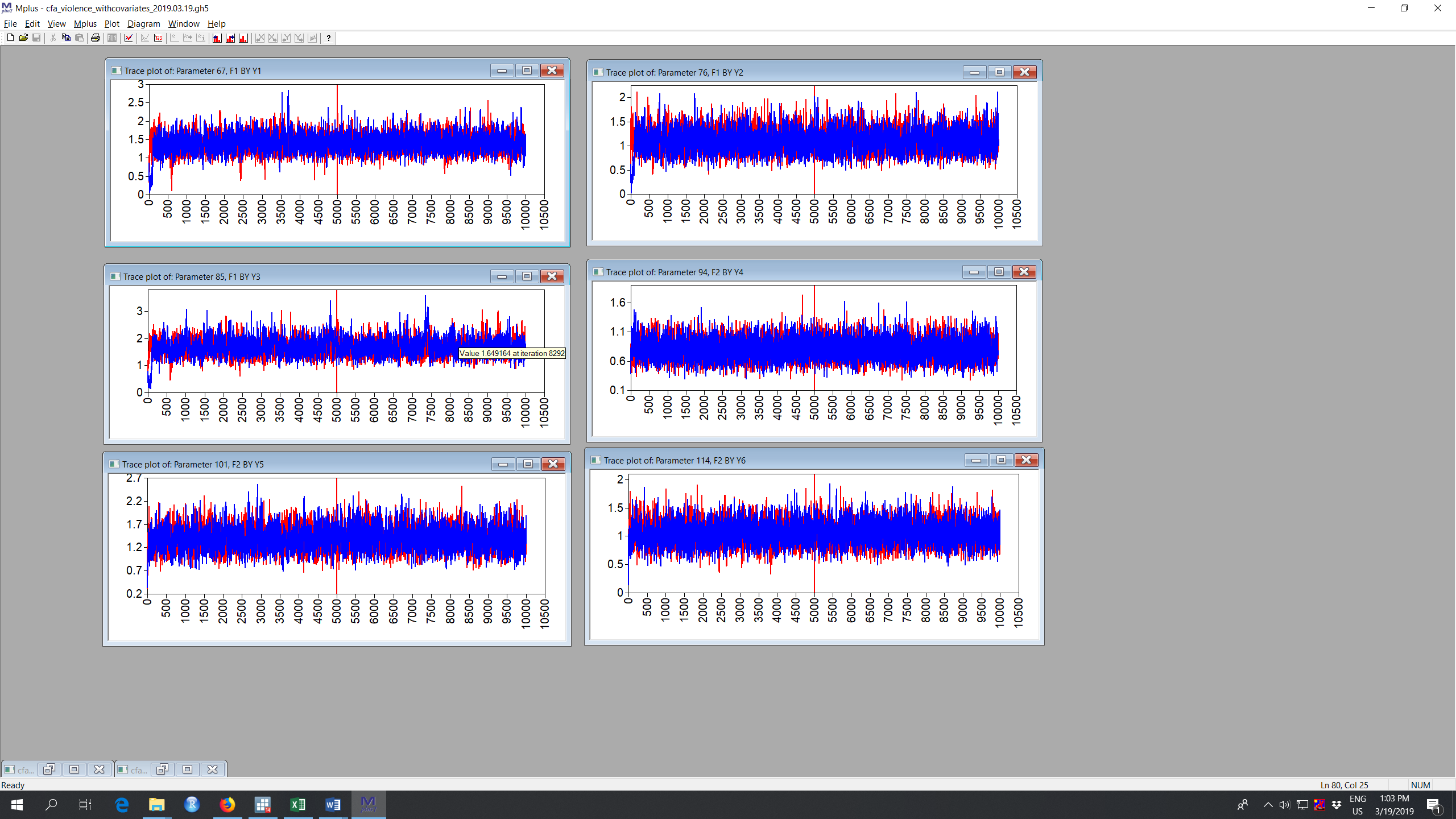


**S1 Fig. DREAMS survey data. Trace plots for the first six hypothesized factor loadings from the Bayesian MIMIC model. They all show “caterpillar”-like behaviour, indicating MCMC sampler is mixing well; efficiently sampling from a maximum in the underlying distribution**

The model fit was assessed using the posterior predictive checks (PPCs). PPCs are obtained by generating replicated data sets from the posterior predictive distribution of the fitted model. These data sets are then compared with the observed data set with respect to the feature of interest by using discrepancy measures [9]. From these PPCs, posterior predictive p-values (PPP-values) can be computed. In this paper the chi-square discrepancy measure [4, 10] was used. Consider data $\boldsymbol{y}_{1},\ldots,\boldsymbol{y}_{n}$ consisting of $n$ random vectors of random variables, given the parameter vector $\boldsymbol{\theta.}$ The chi-square discrepancy is then

$$\chi^{2}\left( \boldsymbol{y;\theta} \right)=\sum_{i=1}^{n} \frac{\left( \boldsymbol{y}_{i}-E\left( \boldsymbol{y}_{i} | \boldsymbol{\theta} \right) \right)^{2}}{Var\left( \boldsymbol{y}_{i} | \boldsymbol{\theta} \right)}.$$

The PPP-value is the proportion of the discrepancy measures obtained from the replicated data that are greater than the discrepancy measure of the observed data. In the M*plus* implementation the PPP-value is computed using every 10^th^ iteration among the iterations used to describe the posterior distribution of parameters. A 95% confidence interval is produced for the difference in the discrepancy measure for the observed and replicated data [4]. If the model is true or close to true, the PPP-value will tend to 0.5 and the discrepancy measure difference will be close to zero falling close to the middle of the confidence interval [4]. The relevant M*plus* codes have been included in the Supporting Information, S2 Appendix.

**References**

1. Gelman A, Hill J: Data analysis using regression and multilevel/hierarchical models: Cambridge university press; 2006.

2. Muthén L, Muthén B: Mplus User’s Guide, 7 edn. Los Angeles, CA: Muthén and Muthén; 1998–2015.

3. Lesaffre E, Lawson AB: Bayesian biostatistics: John Wiley & Sons; 2012.

4. Muthén B, Asparouhov T: Bayesian structural equation modeling: a more flexible representation of substantive theory. *Psychological methods* 2012, 17(3):313.

5. Hayashi K, Marcoulides GA: Examining identification issues in factor analysis. *Structural Equation Modeling* 2006, 13(4):631-645.

6. Gelman A, Jakulin A, Pittau MG, Su Y-S: A weakly informative default prior distribution for logistic and other regression models. *The Annals of Applied Statistics* 2008, 2(4):1360-1383.

7. Asparouhov T, Muthén B: Bayesian analysis of latent variable models using Mplus. *Retrieved June* 2010, 17:2014.

8. Brooks SP, Gelman A: General methods for monitoring convergence of iterative simulations. *Journal of computational and graphical statistics* 1998, 7(4):434-455.

9. Gelman A, Carlin JB, Stern HS, Rubin DB: Bayesian data analysis, 2nd edn. Texts in Statistical Science. In*.*: Boca Raton, London, NewYork, Washington DC: Chapman & Hall, CRC; 2004.

10. Gelman A, Meng X-L, Stern H: Posterior predictive assessment of model fitness via realized discrepancies. *Statistica sinica* 1996:733-760.
