# Supplementary material for: Invariance of the WHO violence against women instrument among Kenyan adolescent girls and young women: Bayesian psychometric modeling
Source: PLoS One. 2021 Oct 15;16(10):e0258651. doi: 10.1371/journal.pone.0258651 (PMC8519454; doi:10.1371/journal.pone.0258651)
Supplement: S1 Table — Distribution of AGYW by Socio-demographics. (DOCX) [file pone.0258651.s002.docx]

**S1 Table.** DREAMS Survey data. Distribution of AGYW by socio-demographics

| Characteristics | Number of AGYW | Percentage of AGYW |
| --- | --- | --- |
| Overall (all AGYW 15-22yrs) | 1,081 | 100.0 |
| Invited to DREAMS |  |  |
| Not-invited | 545 | 50.4 |
| Invited | 536 | 49.6 |
| Site/slum |  |  |
| Korogocho | 617 | 57.1 |
| Viwandani | 464 | 42.9 |
| Age |  |  |
| 15-17yrs | 547 | 50.6 |
| 18-22yrs | 534 | 49.4 |
| Marital/co-habitation status |  |  |
| Never married | 843 | 78.0 |
| Previously married/lived with partner | 33 | 3.1 |
| Currently married/living with partner | 205 | 19.0 |
| Currently in school |  |  |
| No | 455 | 42.1 |
| Yes | 626 | 57.9 |
| Educational level |  |  |
| None/incomplete primary | 124 | 11.5 |
| Complete primary | 217 | 20.1 |
| Incomplete secondary | 491 | 45.4 |
| Complete secondary | 198 | 18.3 |
| Tertiary: university/college/vocational | 51 | 4.7 |
| Religion |  |  |
| Christian | 917 | 84.8 |
| Muslim | 142 | 13.1 |
| Other | 22 | 2.0 |
| Ethnicity |  |  |
| Somali | 90 | 8.3 |
| Kamba | 208 | 19.2 |
| Kikuyu | 319 | 29.5 |
| Kisii | 51 | 4.7 |
| Luhya | 176 | 16.3 |
| Luo | 176 | 16.3 |
| Other | 61 | 5.6 |
| Ever had sex |  |  |
| No | 439 | 40.6 |
| Yes | 642 | 59.4 |
| Sleep hungry at night past 4 weeks |  |  |
| No | 729 | 67.4 |
| Yes | 352 | 32.6 |
| Wealth quantile |  |  |
| Poor | 361 | 33.4 |
| Medium | 360 | 33.3 |
| Wealthy | 360 | 33.3 |
