# Supplementary material for: Invariance of the WHO violence against women instrument among Kenyan adolescent girls and young women: Bayesian psychometric modeling
Source: PLoS One. 2021 Oct 15;16(10):e0258651. doi: 10.1371/journal.pone.0258651 (PMC8519454; doi:10.1371/journal.pone.0258651)
Supplement: S2 Table — Selection of covariates for the direct effect assessment in the A coefficient. Data are LRT p-values. (DOCX) [file pone.0258651.s003.docx]

**S2 Table.** DREAMS survey data. Selection of covariates for the direct effect assessment in the A coefficient. Data are LRT p-values.*

| **Simple univariate model (invited to DREAMS,**  **site and age adjusted)** | Items | | | | | | | | | | | | | | |
| --- | --- | --- | --- | --- | --- | --- | --- | --- | --- | --- | --- | --- | --- | --- | --- |
|  | 1 | 2 | 3 | 4 | 5 | 6 | 7 | 8 | 9 | 10 | 11 | 12 | 13 | 14 | 15 |
| Invited to DREAMS | .994 | .205 | .472 | .029 | .964 | .265 | .759 | .816 | .342 | .623 | .139 | .542 | .684 | .030 | .769 |
| Site | .549 | .298 | .221 | .001 | .008 | .034 | .022 | .003 | .104 | <.001 | .035 | .899 | .174 | .050 | .272 |
| Age | .032 | .014 | .337 | .865 | .001 | .089 | .427 | .354 | .553 | .694 | .270 | .530 | .643 | .046 | .228 |
| Marital status | .032 | .066 | <.001 | .027 | <.001 | .345 | .010 | .011 | .239 | .0177 | .756 | .941 | .987 | .598 | .613 |
| Currently in school | .681 | .008 | .204 | .108 | <.001 | .217 | .018 | .006 | .002 | .007 | .234 | .775 | .013 | <.001 | .120 |
| Education level | .286 | .456 | .244 | .889 | <.001 | .262 | .240 | .006 | .3745 | .6152 | .705 | .439 | .213 | .780 | .848 |
| Religion | <.001 | .003 | <.001 | .074 | .035 | .024 | .304 | .068 | .0731 | .0561 | .796 | .001 | <.001 | .812 | .110 |
| Ethnicity | .013 | .031 | <.001 | .449 | .080 | .133 | .469 | .257 | .8981 | .2691 | .610 | .031 | .007 | .541 | .412 |
| Ever had sex | .013 | .004 | .004 | .007 | <.001 | .015 | .004 | .001 | .011 | .003 | .395 | <.001 | <.001 | <.001 | <.001 |
| Slept hungry at night in past 4 weeks | <.001 | <.001 | .046 | .021 | .012 | .021 | .040 | .027 | .925 | .009 | .300 | .101 | <.001 | .001 | <.001 |
| Wealth quantile | .548 | .205 | .601 | .175 | .176 | .181 | .598 | .440 | .501 | .012 | .768 | .369 | .060 | .013 | .012 |
| **Multiple univariate model (fully adjusted)** | | | | | | | | | | | | | | | |
| Invited to DREAMS | .589 | .518 | .420 | .061 | .424 | .288 | .936 | .631 | .554 | .473 | .139 | .764 | .610 | .056 | .671 |
| Site | .591 | .804 | .569 | .007 | .020 | .038 | .087 | .033 | .108 | <.001 | .035 | .849 | .397 | .990 | .400 |
| Age | .334 | .524 | .931 | .146 | .841 | .010 | .170 | .885 | .024 | .072 | .270 | .068 | .024 | .080 | .182 |
| Marital status | .034 | .028 | <.001 | .105 | .018 |  | .182 | .474 |  | .064 |  |  |  |  |  |
| Currently in school |  | .080 |  |  | .871 |  | .453 | .512 | .035 | .218 |  |  | .745 | .532 |  |
| Education level |  |  |  |  | .019 |  |  | .073 |  |  |  |  |  |  |  |
| Religion | .168 | .653 | .529 | .806 | .522 | .041 |  | .131 | .179 | .229 |  | .076 | .192 |  |  |
| Ethnicity | .405 | .416 | .559 | .886 | .755 |  |  |  |  |  |  | .516 | .764 |  |  |
| Ever had sex | .055 | .102 | .059 | .057 | .023 | .055 | .138 | .271 | .407 | .165 |  | <.001 | <.001 | <.001 | <.001 |
| Slept hungry at night in past 4 weeks | <.001 | <.001 | .130 | .024 | .096 | .031 | .068 | .092 |  | .028 |  | .155 | .001 | .011 | <.001 |
| Wealth quantile |  |  |  |  |  |  |  |  |  | .016 |  |  | .122 | .083 | .164 |

*Only covariates with LRT p≤0.05 in the multivariable univariate model were included in the A part of MIMIC model in equation 1 for assessment of (non)invariance. Invitation to DREAMS, site, and age were included even if they were not significant as they were of interest to the research, but we also wished to correct for their impact. All covariates were included in the B part of the MIMIC model in equation 1.
