# Supplementary material for: Invariance of the WHO violence against women instrument among Kenyan adolescent girls and young women: Bayesian psychometric modeling
Source: PLoS One. 2021 Oct 15;16(10):e0258651. doi: 10.1371/journal.pone.0258651 (PMC8519454; doi:10.1371/journal.pone.0258651)
Supplement: S3 Table — Bayesian MIMIC model: Confirmatory Factor Analysis. (DOCX) [file pone.0258651.s004.docx]

**S3 Table.** DREAMS survey data. Bayesian MIMIC model: Confirmatory Factor Analysis

| Item | Factor loading (95% Credibility Intervals) | | | | |
| --- | --- | --- | --- | --- | --- |
|  | Psychological | Physical | Sexual | |  |
| 1. Say or do something to humiliate you in front of others? | **0.915 (0.737-1.097)*** | -0.046 (-0.190-0.102) | | -0.038 (-0.187-0.103) | |
| 2. Threaten to hurt or harm you or someone close to you? | **0.729 (0.530-0.942)*** | 0.081 (-0.079-0.235) | | 0.079 (-0.071-0.220) | |
| 3. Insult you or make you feel bad about yourself? | **0.903 (0.735-1.077)*** | 0.026 (-0.110-0.163) | | 0.006 (-0.131-0.140) | |
| 4 Push you, shake you, or throw something at you? | 0.201 (0.042-0.356) | **0.575 (0.390-0.757)*** | | 0.033 (-0.108-0.169) | |
| 5. Slap you? | 0.002 (-0.146-0.148) | **0.820 (0.646-0.983)*** | | -0.013 (-0.146-0.120) | |
| 6. Twist your arm or pull your hair? | 0.017 (-0.139-0.172) | **0.710 (0.522-0.884*)** | | 0.097 (-0.051-0.238) | |
| 7. Punch you with his fist or something that could hurt you? | -0.027 (-0.148-0.088) | **0.962 (0.825-1.087)*** | | -0.030 (-0.147-0.088) | |
| 8. Kick you, drag you, or beat you up? | -0.025 (-0.132-0.077) | **0.967 (0.844-1.082)*** | | -0.007 (-0.111-0.096) | |
| 9. Try to choke you or burn you on purpose? | 0.002 (-0.144-0.152) | **0.841 (0.632-1.007)*** | | -0.020 (-0.172-0.131) | |
| 10. Threatened to attack you with a knife or other weapon? | 0.098 (-0.048-0.248) | **0.626 (0.42-0.809)*** | | 0.018 (-0.116-0.157) | |
| 11. Attacked you with a weapon? | -0.057 (-0.233-0.125) | **0.716 (0.463-0.922)*** | | 0.056 (-0.120-0.235) | |
| 12. Touched you in a sexual way (e.g. kissing, grabbing, or fondling), when you did not want them to? | 0.066 (-0.058-0.193) | 0.033 (-0.088-0.153) | | **0.828 (0.693-0.957)*** | |
| 13. Try to have sexual intercourse with you when you did not want to but did not succeed? | -0.010 (-0.121-0.098) | 0.027 (-0.081-0.132) | | **0.922 (0.804-1.039)*** | |
| 14. Physically forced you to have sexual intercourse even when you did not want to? | -0.027 (-0.118-0.058) | -0.018 (-0.103-0.065) | | **0.936 (0.833-1.039)*** | |
| 15. Forced you to perform sexual acts when you did not want to? | 0.011 (-0.079-0.103) | -0.009 (-0.098-0.078) | | **0.943 (0.834-1.045)*** | |
| **Factor Correlations** |  |  | |  | |
| Psychological | 1.000 |  | |  | |
| Physical | 0.782 (0.634-0.912) | 1.000 | |  | |
| Sexual | 0.617 (0.442-0.769) | 0.632 (0.477-0.764) | | 1.000 | |

Bolden values indicate hypothesized major loadings; * indicates values are significant at 5% level; MIMIC, Multiple Indicator Multiple Causes.
