# Supplementary material for: Invariance of the WHO violence against women instrument among Kenyan adolescent girls and young women: Bayesian psychometric modeling
Source: PLoS One. 2021 Oct 15;16(10):e0258651. doi: 10.1371/journal.pone.0258651 (PMC8519454; doi:10.1371/journal.pone.0258651)
Supplement: S4 Table — (DOCX) [file pone.0258651.s005.docx]

**S4 Table.** Bayesian MIMIC model: Effect of Covariates on Factors (B-coefficients).

|  | B-coefficients (95% Credibility Intervals) | | |
| --- | --- | --- | --- |
| Covariates | Psychological | Physical | Sexual |
| Invited to DREAMS (Ref: not invited) | -0.005 (-0.341-0.341) | -0.021 (-0.224-0.191) | -0.088 (-0.275-0.121) |
| Site/slum (Ref: Korogocho) | -0.006 (-0.367-0.320) | -0.207 (-0.412-0.028) | 0.011 (-0 .194-0.228) |
| Age (Ref: 15-17yrs) | 0.011 (-0.315-0.329) | -0.097 (-0.322-0.122) | -0.133 (-0.355-0.088) |
| Marital/co-habitation status (Ref: never married) | |  |  |
| Previously married/lived with partner | 0.090 (-0.040-0.217) | 0.071 (-0.006-0.148) | -0.067 (-0.151-0.014) |
| Currently married/living with partner | -0.184 (-0.405-0.047) | -0.020 (-0.137-0.101) | -0.096 (-0.201-0.006) |
| Currently in school (no/yes. Ref: no) | -0.059 (-0.163-0.045) | -0.09 (-0.207-0.027) | -0.017 (-0.125-0.094) |
| Educational level (Ref: None/ incomplete primary) |  |  |  |
| Complete primary | -0.103 (-0.210-0.009) | -0.103 (-0.218-0.016) | -0.133 (-0.245--0.015)* |
| Incomplete secondary | 0.022 (-0.100-0.151) | -0.134 (-0.270-0.002) | -0.076 (-0.209-0.062) |
| Complete secondary | -0.044 (-0.159-0.076) | -0.167 (-0.302--0.035)* | -0.036 (-0.159-0.096) |
| Tertiary: university/college/vocational | -0.045 (-0.133-0.045) | -0.109 (-0.222-0.001) | 0.032 (-0.059-0.123) |
| Religion (Ref: Muslim) |  |  |  |
| Christian | 0.208 (-0.009-0.421) | 0.196 (-0.038-0.435) | 0.578 (0.236-0.927)* |
| Other | 0.096 (-0.016-0.203) | 0.101 (-0.017-0.218) | 0.25 (0.093-0.399)* |
| Ethnicity (Ref: Somali) |  |  |  |
| Kamba | 0.079 (-0.197-0.354) | -0.137 (-0.428-0.15) | -0.535 (-0.956--0.134)* |
| Kikuyu | 0.050 (-0.259-0.369) | -0.153 (-0.481-0.180) | -0.59 (-1.071--0.143)* |
| Kisii | 0.052 (-0.112-0.211) | -0.029 (-0.202-0.150) | -0.305 (-0.542--0.082)* |
| Luhya | 0.021 (-0.234-0.278) | -0.159 (-0.427-0.115) | -0.481 (-0.873--0.115)* |
| Luo | 0.062 (-0.191-0.319) | -0.081 (-0.347-0.189) | -0.458 (-0.845--0.087)* |
| Other | 0.002 (-0.122-0.127) | -0.049 (-0.174-0.072) | -0.266 (-0.509--0.062)* |
| Ever had sex (no/yes. Ref: no) | 0.133 (0.015-0.246)* | 0.09 (-0.043-0.221)* | 0.372 (0.177-0.551)* |
| Slept hungry at night past 4 weeks (no/yes. Ref: no) | 0.066 (-0.027-0.158) | 0.052 (-0.036-0.142) | 0.052 (-0.059-0.165) |
| Wealth quantile (Ref: Poor) |  |  |  |
| Medium | -0.010 (-0.099-0.083) | -0.077 (-0.174-0.022) | -0.103 (-0.204--0.001)* |
| Wealthy | 0.011 (-0.095-0.119) | 0.002 (-0.118-0.12) | -0.060 (-0.180-0.059) |
