# Supplementary material for: Invariance of the WHO violence against women instrument among Kenyan adolescent girls and young women: Bayesian psychometric modeling
Source: PLoS One. 2021 Oct 15;16(10):e0258651. doi: 10.1371/journal.pone.0258651 (PMC8519454; doi:10.1371/journal.pone.0258651)
Supplement: S5 Table — (DOCX) [file pone.0258651.s006.docx]

**S4 Table.** Bayesian MIMIC model: Effect of Covariates on 15 experience of violence Items (A-coefficients).

|  | A-coefficients | | |
| --- | --- | --- | --- |
| Covariates | 1 | 2 | 3 |
| Invited to DREAMS (Ref: not invited) | -0.039 (-0.374-0.275) | 0.024 (-0.246-0.284) | 0.012 (-0.305-0.323) |
| Site/slum (Ref: Korogocho) | 0.02 (-0.292-0.353) | -0.008 (-0.261-0.266) | 0.035 (-0.264-0.356) |
| Age (Ref: 15-17yrs) | 0.033 (-0.27-0.334) | 0.066 (-0.189-0.315) | -0.031 (-0.329-0.260) |
| Marital/co-habitation status (Ref: never married) | | | |
| Previously married/lived with partner | -0.038 (-0.150-0.070) | -0.032 (-0.130-0.063) | 0.016 (-0.089-0.122) |
| Currently married/living with partner | 0.047 (-0.161-0.264) | 0.006 (-0.166-0.198) | 0.05 (-0.148-0.253) |
| Currently in school (no/yes. Ref: no) |  |  |  |
| Educational level (Ref: None/ Incomplete primary) | | |  |
| Complete primary |  |  |  |
| Incomplete secondary |  |  |  |
| Complete secondary |  |  |  |
| Tertiary: university/college/vocational |  |  |  |
| Religion (Ref: Muslim) |  |  |  |
| Christian |  |  |  |
| Other |  |  |  |
| Ethnicity (Ref: Somali) |  |  |  |
| Kamba |  |  |  |
| Kikuyu |  |  |  |
| Kisii |  |  |  |
| Luhya |  |  |  |
| Luo |  |  |  |
| Other |  |  |  |
| Ever had sex (no/yes. Ref: no) |  |  |  |
| Slept hungry at night past 4 weeks (no/yes. Ref: no) | 0.090 (0.001-0.180)* | 0.100 (0.009-0.191)* |  |
| Wealth quantile (Ref: Poor) |  |  |  |
| Medium |  |  |  |
| Wealthy |  |  |  |

**Table S4.** Continued

|  | A-coefficients | | |
| --- | --- | --- | --- |
| Covariates | 4 | 5 | 6 |
| Invited to DREAMS (Ref: not invited) | 0.095 (-0.068-0.262) | 0.040 (-0.141-0.221) | 0.056 (-0.118-0.228) |
| Site/slum (Ref: Korogocho) | -0.046 (-0.215-0.120) | 0.024 (-0.161-0.215) | 0.023 (-0.153-0.207) |
| Age (Ref: 15-17yrs) | -0.038 (-0.200-0.129) | 0.057 (-0.130-0.256) | -0.108 (-0.272-0.069) |
| Marital/co-habitation status (Ref: never married) | |  |  |
| Previously married/lived with partner |  | 0.03 (-0.036-0.098) |  |
| Currently married/living with partner |  | 0.116 (0.010-0.22)* |  |
| Currently in school (no/yes. Ref: no) |  |  |  |
| Educational level (Ref: None/some primary) |  |  |  |
| Complete primary |  | -0.068 (-0.172-0.033) |  |
| Some secondary |  | -0.135 (-0.255--0.018)* | |
| Complete secondary |  | -0.062 (-0.18-0.057) |  |
| Tertiary: university/college/vocational |  | -0.036 (-0.132-0.060) |  |
| Religion (Ref: Muslim) |  |  |  |
| Christian |  |  | 0.058 (-0.066-0.187) |
| Other |  |  | -0.057 (-0.157-0.035) |
| Ethnicity (Ref: Somali) |  |  |  |
| Kamba |  |  |  |
| Kikuyu |  |  |  |
| Kisii |  |  |  |
| Luhya |  |  |  |
| Luo |  |  |  |
| Other |  |  |  |
| Ever had sex (no/yes. Ref: no) |  | 0.071 (-0.046-0.189) |  |
| Slept hungry at night past 4 weeks (no/yes. Ref: no) | 0.032 (-0.060-0.121) |  | 0.029 (-0.065-0.123) |
| Wealth quantile (Ref: Poor) |  |  |  |
| Medium |  |  |  |
| Wealthy |  |  |  |

**Table S4.** Continued

|  | A-coefficients | | |
| --- | --- | --- | --- |
| Covariates | 7 | 8 | 9 |
| Invited to DREAMS (Ref: not invited) | -0.019 (-0.222-0.183) | 0.012 (-0.184-0.212) | -0.055 (-0.262-0.158) |
| Site/slum (Ref: Korogocho) | 0.050 (-0.159-0.267) | -0.002 (-0.218-0.202) | 0.044 (-0.176-0.260) |
| Age (Ref: 15-17yrs) | 0.032 (-0.166-0.242) | 0.039 (-0.163-0.241) | -0.159 (-0.373-0.068) |
| Marital/co-habitation status (Ref: never married) | |  |  |
| Previously married/lived with partner |  |  |  |
| Currently married/living with partner |  |  |  |
| Currently in school (no/yes. Ref: no) |  |  | -0.139 (-0.305-0.025) |
| Educational level (Ref: None/some primary) | |  |  |
| Complete primary |  |  |  |
| Some secondary |  |  |  |
| Complete secondary |  |  |  |
| Tertiary: university/college/vocational | |  |  |
| Religion (Ref: Muslim) |  |  |  |
| Christian |  |  |  |
| Other |  |  |  |
| Ethnicity (Ref: Somali) |  |  |  |
| Kamba |  |  |  |
| Kikuyu |  |  |  |
| Kisii |  |  |  |
| Luhya |  |  |  |
| Luo |  |  |  |
| Other |  |  |  |
| Ever had sex (no/yes. Ref: no) |  |  |  |
| Slept hungry at night past 4 weeks (no/yes. Ref: no) |  |  |  |
| Wealth quantile (Ref: Poor) |  |  |  |
| Medium |  |  |  |
| Wealthy |  |  |  |

**Table S4.** Continued

|  | A-coefficients | | |
| --- | --- | --- | --- |
| Covariates | 10 | 11 | 12 |
| Invited to DREAMS (Ref: not invited) | -0.047 (-0.214-0.119) | -0.134 (-0.351-0.086) | 0.036 (-0.138-0.201) |
| Site/slum (Ref: Korogocho) | -0.244 (-0.446--0.038)* | -0.074 (-0.309-0.162) | 0.026 (-0.154-0.195) |
| Age (Ref: 15-17yrs) | -0.028 (-0.193-0.144) | 0.093 (-0.118-0.305) | -0.023 (-0.201-0.16) |
| Marital/co-habitation status (Ref: never married) |  |  |  |
| Previously married/lived with partner |  |  |  |
| Currently married/living with partner |  |  |  |
| Currently in school (no/yes. Ref: no) |  |  |  |
| Educational level (Ref: None/some primary) |  |  |  |
| Complete primary |  |  |  |
| Some secondary |  |  |  |
| Complete secondary |  |  |  |
| Tertiary: university/college/vocational |  |  |  |
| Religion (Ref: Muslim) |  |  |  |
| Christian |  |  |  |
| Other |  |  |  |
| Ethnicity (Ref: Somali) |  |  |  |
| Kamba |  |  |  |
| Kikuyu |  |  |  |
| Kisii |  |  |  |
| Luhya |  |  |  |
| Luo |  |  |  |
| Other |  |  |  |
| Ever had sex (no/yes. Ref: no) |  |  | -0.020 (-0.189-0.143) |
| Slept hungry at night past 4 weeks (no/yes. Ref: no) | 0.068 (-0.041-0.176) |  |  |
| Wealth quantile (Ref: Poor) |  |  |  |
| Medium | -0.215 (-0.363--0.076)* |  |  |
| Wealthy | 0.033 (-0.108-0.167) |  |  |

**Table S4.** Continued

|  | A-coefficients | | |
| --- | --- | --- | --- |
| Covariates | 13 | 14 | 15 |
| Invited to DREAMS (Ref: not invited) | 0.041 (-0.144-0.215) | -0.066 (-0.25-0.109) | 0.015 (-0.17-0.196) |
| Site/slum (Ref: Korogocho) | -0.013 (-0.201-0.167) | -0.039 (-0.226-0.14) | 0.03 (-0.159-0.214) |
| Age (Ref: 15-17yrs) | -0.036 (-0.223-0.155) | -0.001 (-0.188-0.191) | -0.007 (-0.196-0.186) |
| Marital/co-habitation status (Ref: never married) |  |  |  |
| Previously married/lived with partner |  |  |  |
| Currently married/living with partner |  |  |  |
| Currently in school (no/yes. Ref: no) |  |  |  |
| Educational level (Ref: None/some primary) |  |  |  |
| Complete primary |  |  |  |
| Some secondary |  |  |  |
| Complete secondary |  |  |  |
| Tertiary: university/college/vocational |  |  |  |
| Religion (Ref: Muslim) |  |  |  |
| Christian |  |  |  |
| Other |  |  |  |
| Ethnicity (Ref: Somali) |  |  |  |
| Kamba |  |  |  |
| Kikuyu |  |  |  |
| Kisii |  |  |  |
| Luhya |  |  |  |
| Luo |  |  |  |
| Other |  |  |  |
| Ever had sex (no/yes. Ref: no) | -0.034 (-0.211-0.138) | 0.098 (-0.079-0.276) | 0.001 (-0.178-0.172) |
| Slept hungry at night past 4 weeks (no/yes. Ref: no) | 0.072 (-0.028-0.169) | 0.073 (-0.036-0.183) | 0.139 (0.028-0.251)* |
| Wealth quantile (Ref: Poor) |  |  |  |
| Medium |  |  |  |
| Wealthy |  |  |  |
